# Supplementary material for: Mitochondrial movement during its association with chloroplasts in Arabidopsis thaliana
Source: Commun Biol. 2021 Mar 5;4:292. doi: 10.1038/s42003-021-01833-8 (PMC7935954; doi:10.1038/s42003-021-01833-8)
Supplement: Supplementary file 2 — Supplementary Information [file 42003_2021_1833_MOESM2_ESM.pdf]

Supplementary information

Oikawa, K. *et al.*

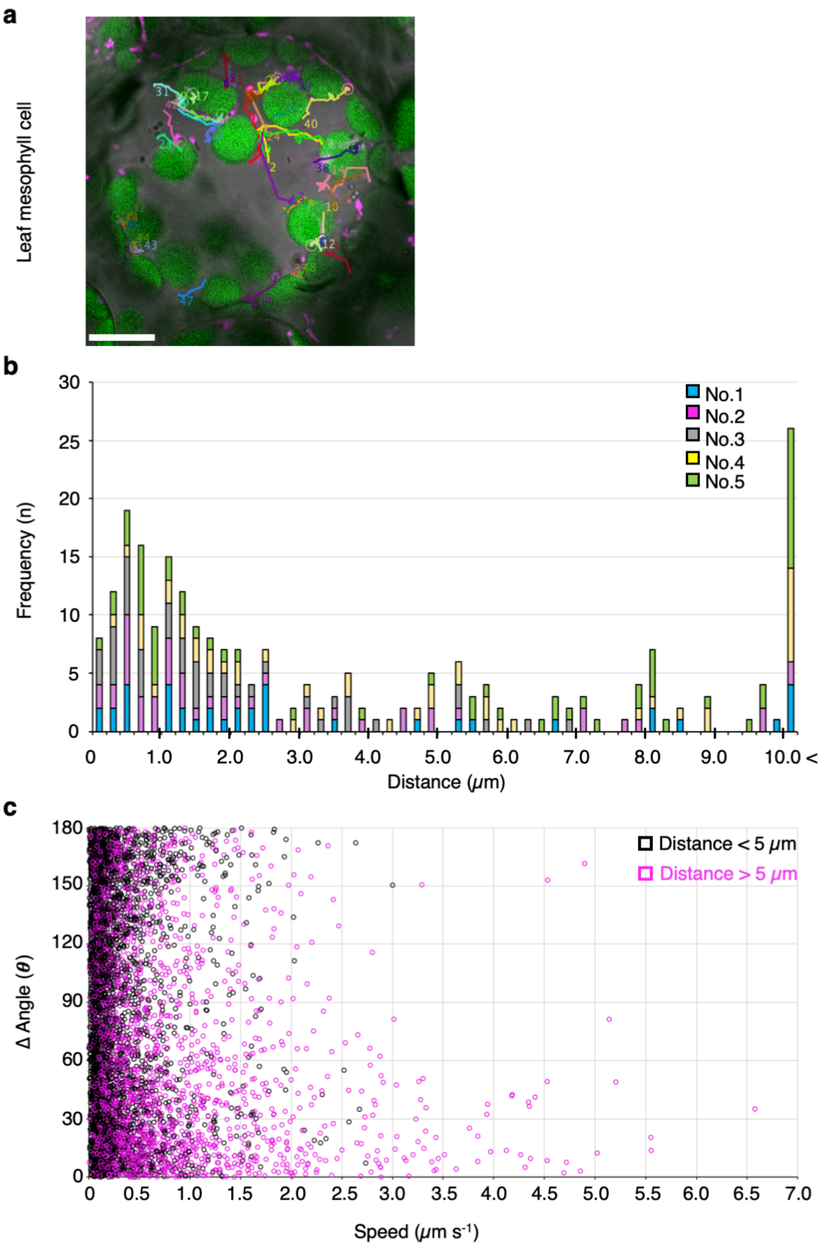

Supplementary Fig. 1 Characterization of mitochondrial movements in leaf mesophyll cells. a Representative image of the trajectories of mitochondrial movements acquired from time-lapse analysis of images of leaf palisade mesophyll cells taken for 30 s at 250-ms intervals. Scale bar; 10 µm. **b** Distribution of the distance between the first and last points in trajectories of mitochondrial movements (leaf cell No. 1, blue (n=34); leaf cell No. 2, magenta (n=45); leaf cell No. 3, gray (n=57); leaf cell No. 4, yellow (n=46), and leaf cell No. 5, green (n=64)). **c** Scatter plot of speed (x-axis) and angle changes (y-axis) of mitochondria at each time point acquired from the trajectories of mitochondrial movement, which are separated into migration distances shorter (open circles) or longer (filled, magenta circles) than 5.0 µm in (**b**).

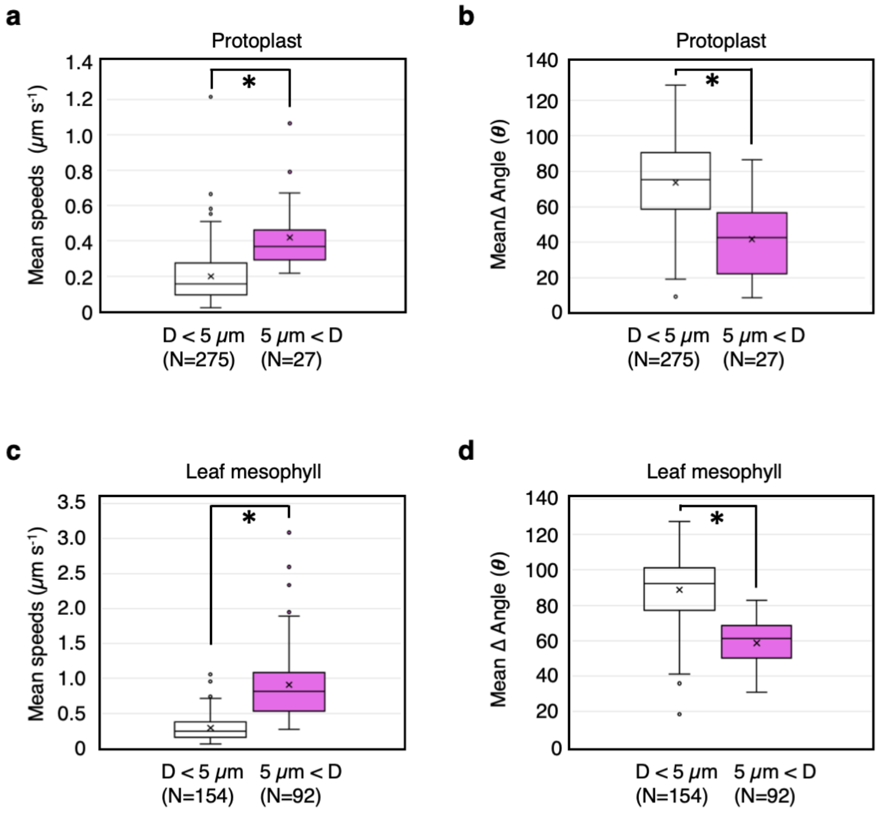

Supplementary Fig. 2 Comparison of mean speeds and angle change of mitochondria between short- or long-distance migration. a,c Mean speed of mitochondria from both mitochondria in short (D < 5µm)- and long (5µm < D)- distance migration in protoplasts (**a**) and leaf mesophyll cells (**c**). **b,d** Mean angle change (Δ Angle (***θ***)) of mitochondria from both mitochondria in short (D < 5µm)- and long (5µm < D)- distance migration in protoplasts (**b**) and leaf mesophyll cells (**d**). *P<0.01 (Student's-*t* test; 3.39E-06 in (**a**), 9.01E-08 in (**b**), 7.39E-20 in (**c**), and 2.26E-22 in (**d**)).

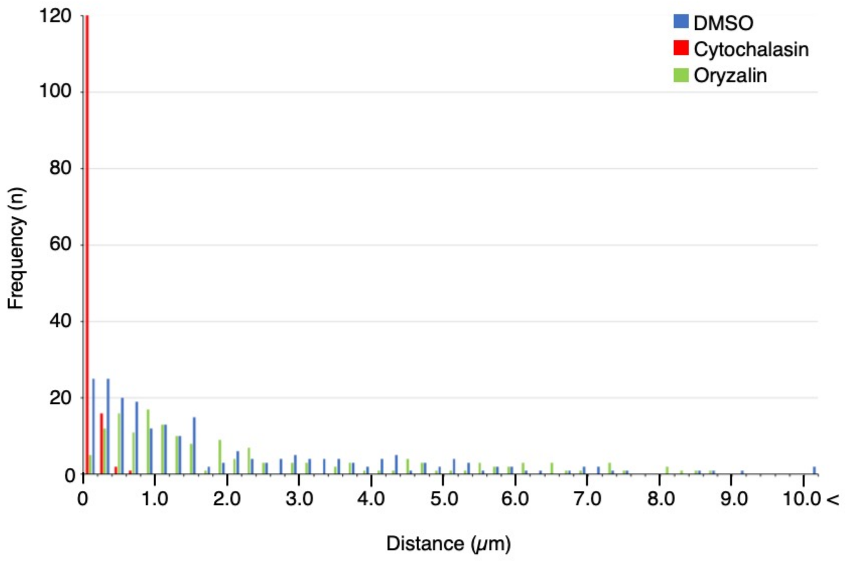

Supplementary Fig. 3 Effect of cytoskeletal inhibitors on the distance of mitochondrial migration. Distribution of the distance between the first and last points in trajectories of mitochondrial movement. DMSO (n=209), blue; cytochalasin B (n=181), red; and oryzalin (n=200), green.

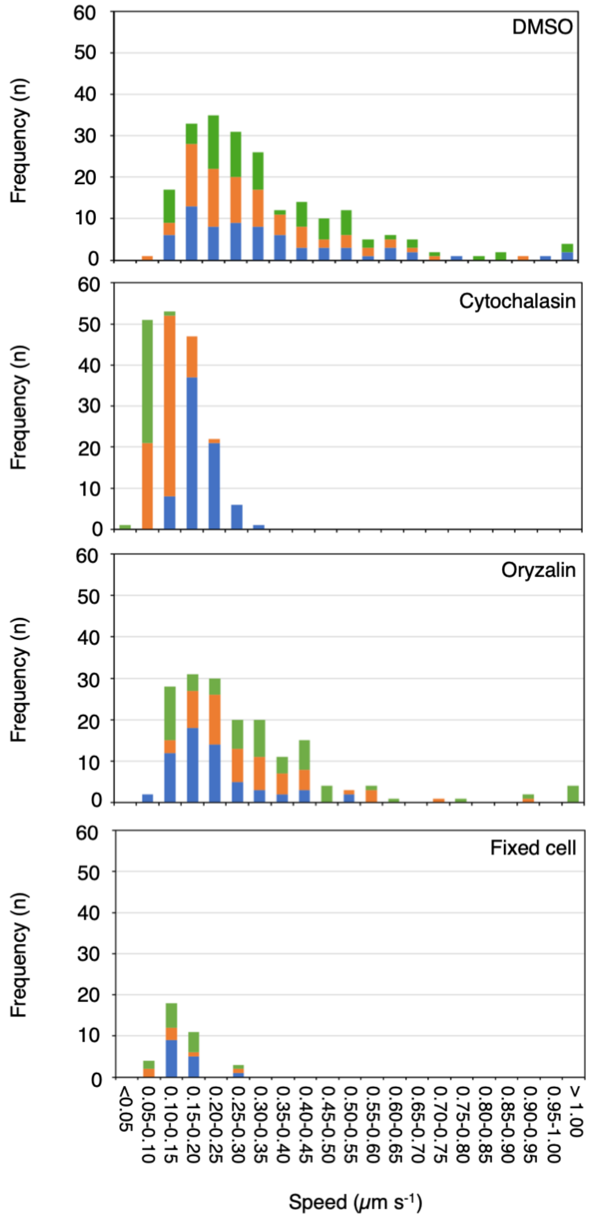

Supplementary Fig. 4 Effects of cytoskeletal inhibitors on the speed distribution of mitochondrial movement. Speed distribution of mitochondrial movement from three independent protoplasts (protoplast No. 1, blue; protoplast No. 2, orange; and protoplast No. 3, green) treated with DMSO (n=209), cytochalasin B (an F-actin-disrupting drug) (n=181), and oryzalin (a microtubule-disrupting drug) (n=200). Thirty-six mitochondria from three independent protoplasts were analyzed in fixed cells.

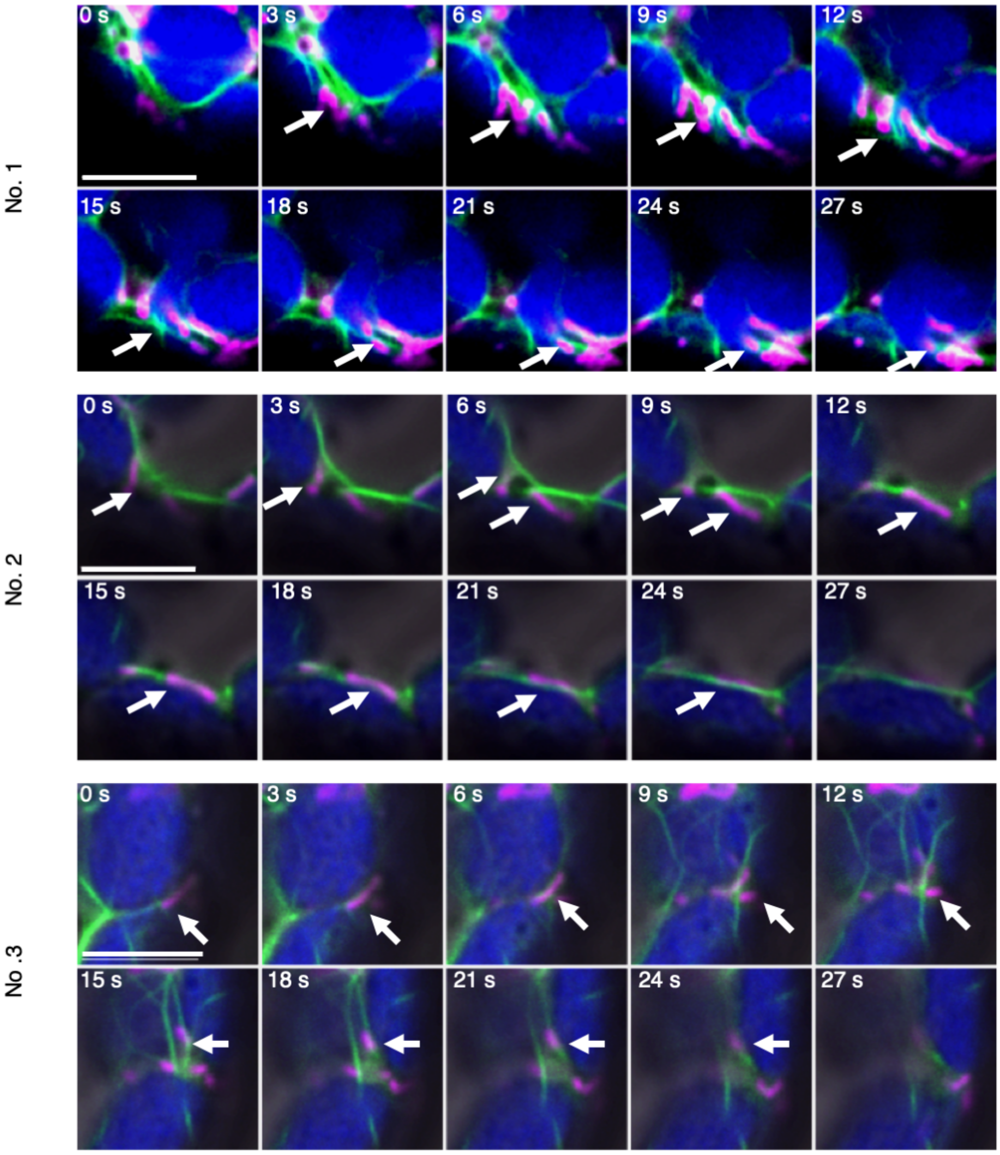

Supplementary Fig. 5 Association of mitochondria and F-actin on chloroplasts. Representative images are acquired from time-lapse analysis of three protoplasts (No.1-3) expressing both the pGWT35S-Lifeact-Citrine and MT-RFP for visualizing F-actin (green) and mitochondrion (magenta). The interaction between mitochondria, F-actin, and chloroplasts (blue) are shown (white arrows). Scale bars: 5 µm.

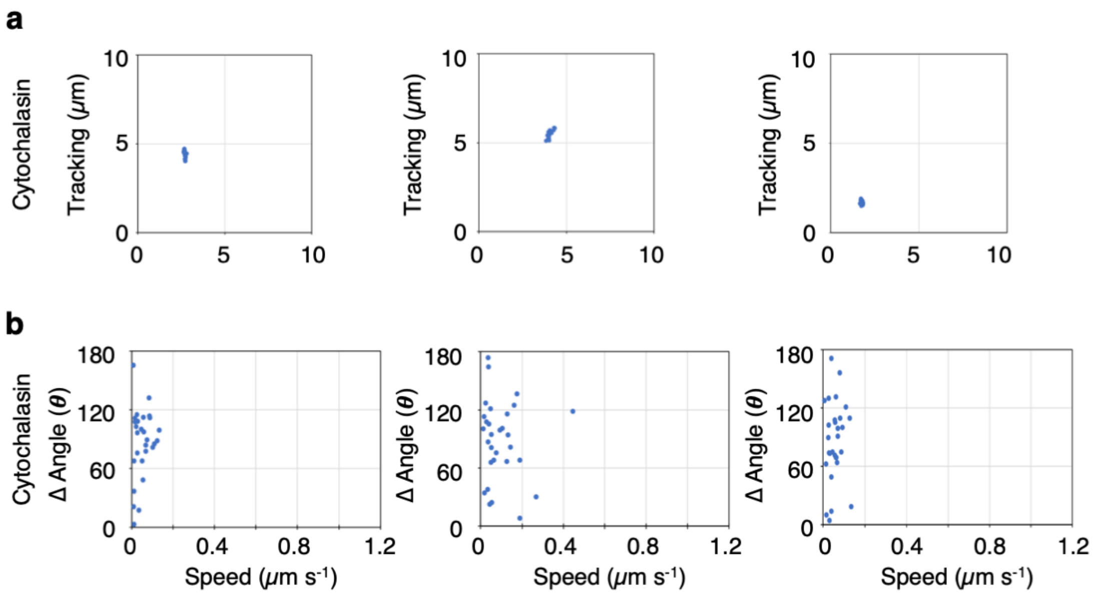

Supplementary Fig. 6 Characterization of mitochondrial movements in cytochalasin-treated protoplasts expressing Lifeact-Citrine gene. a, **b** Representative trajectories (**a**) and scatter plot of speed and angle changes (**b**) of three movements of mitochondria obtained from three protoplasts treated with 500 μM cytochalasin.

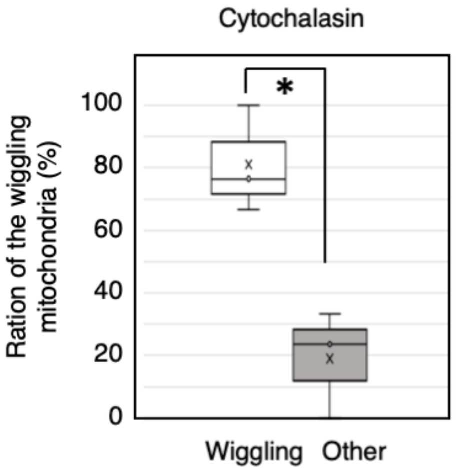

Supplementary Fig. 7 Number of wiggling mitochondria in F-actin-disrupted protoplasts. Ratio of number of wiggling mitochondria with chloroplast to total number of mitochondria in cytochalasin-treated protoplasts expressing pGWT35S-Lifeact-Citrine gene. *P < 0.01 (Student's *t* test).

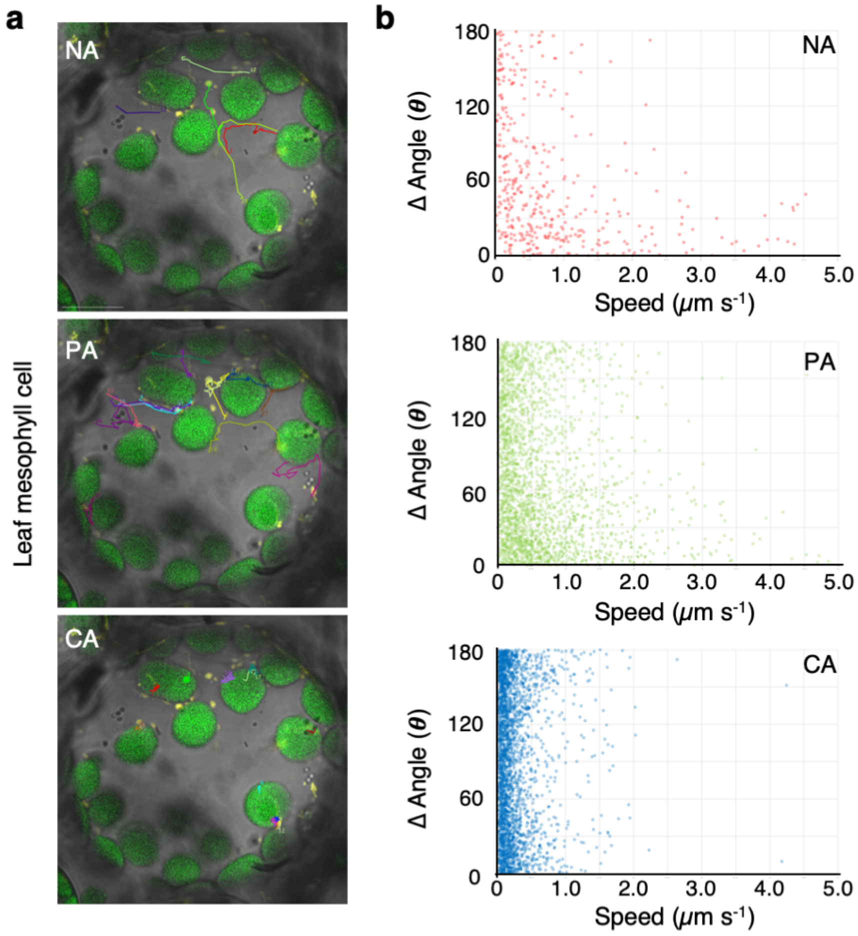

Supplementary Fig. 8 Mitochondrial movements in leaf mesophyll cells are influenced by their association with chloroplasts. a Two representative images of three types of mitochondrial movements: no association (NA), partial association (PA), and continuous association (CA) with chloroplasts. Trajectories of mitochondrial movements were constructed from time-lapse analysis of images acquired for 30 s at 250-ms intervals. Scale bar: 10 µm. **b** Scatter plot of speed (x-axis) and angle changes (y-axis) of three different types of mitochondrial movements: NA (red), PA (green), and CA (blue) at each time point acquired from the trajectories of mitochondrial movements in three different protoplasts.

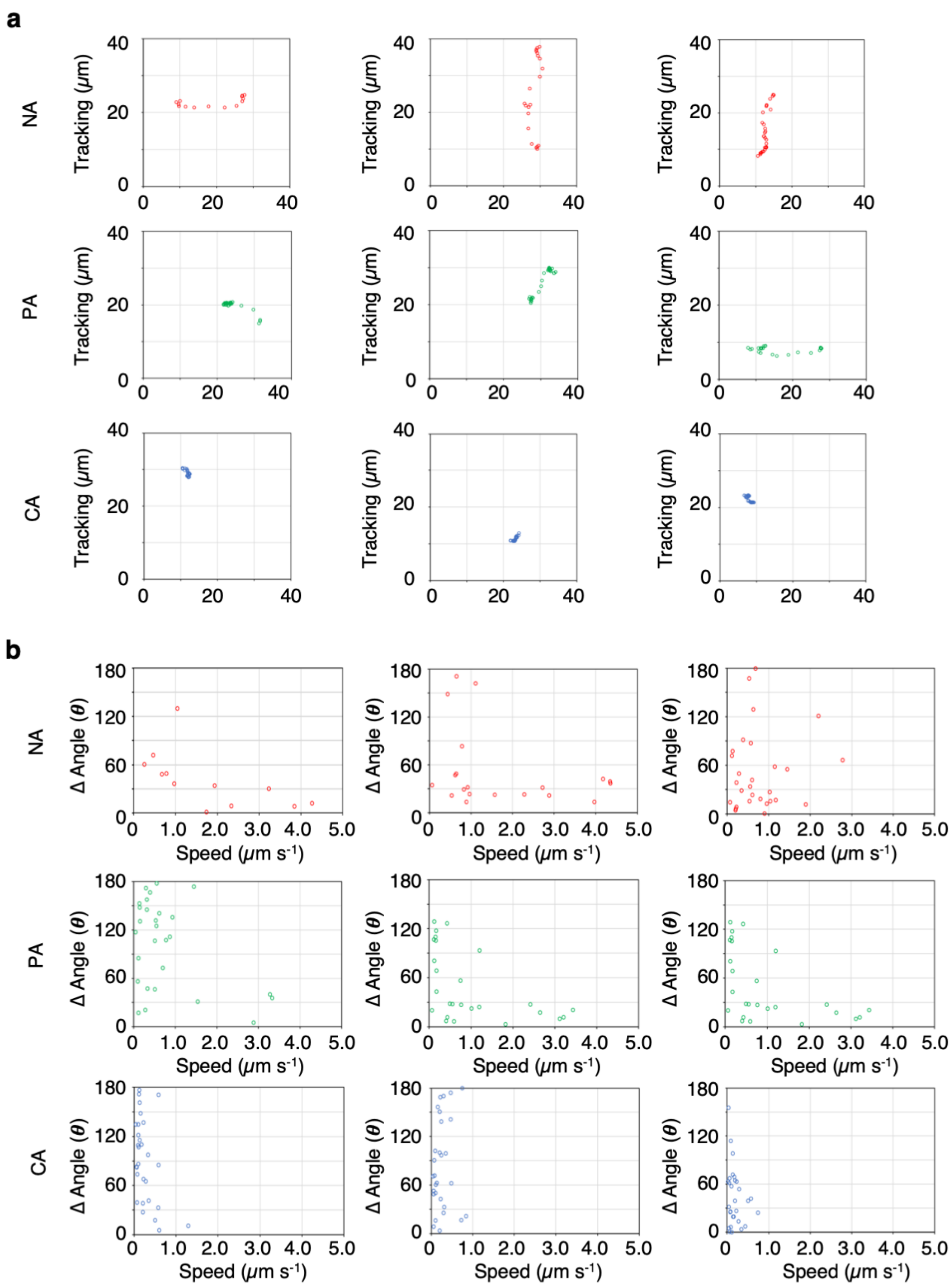

Supplementary Fig. 9 Characterization of three distinct movements of mitochondria associated with chloroplast in leaf mesophyll cells. a, b Representative trajectories (**a**) and scatter plots of speed and angle changes (**b**) of three mitochondrial movements were classified as no association, NA (upper panels); partial association, PA (middle panels); and continuous association, CA (lower panels) with chloroplasts in leaf mesophyll cells.

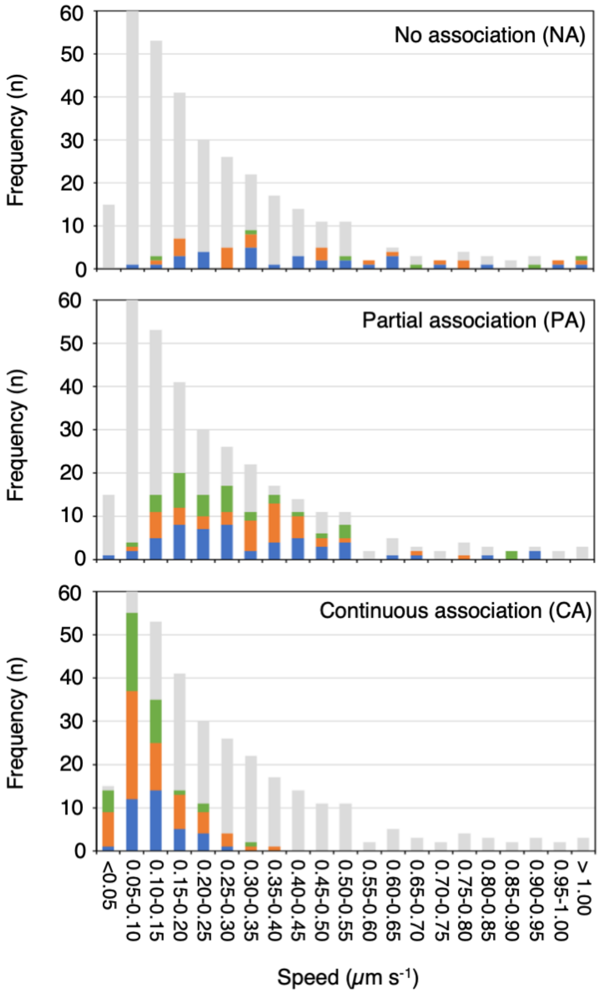

Supplementary Fig. 10 Relationship between mitochondrial speed and association with chloroplasts. Frequency of speeds among mitochondria with no association (NA, n=58), partial association (PA, n=138), and continuous association (CA, n=139) with chloroplasts.

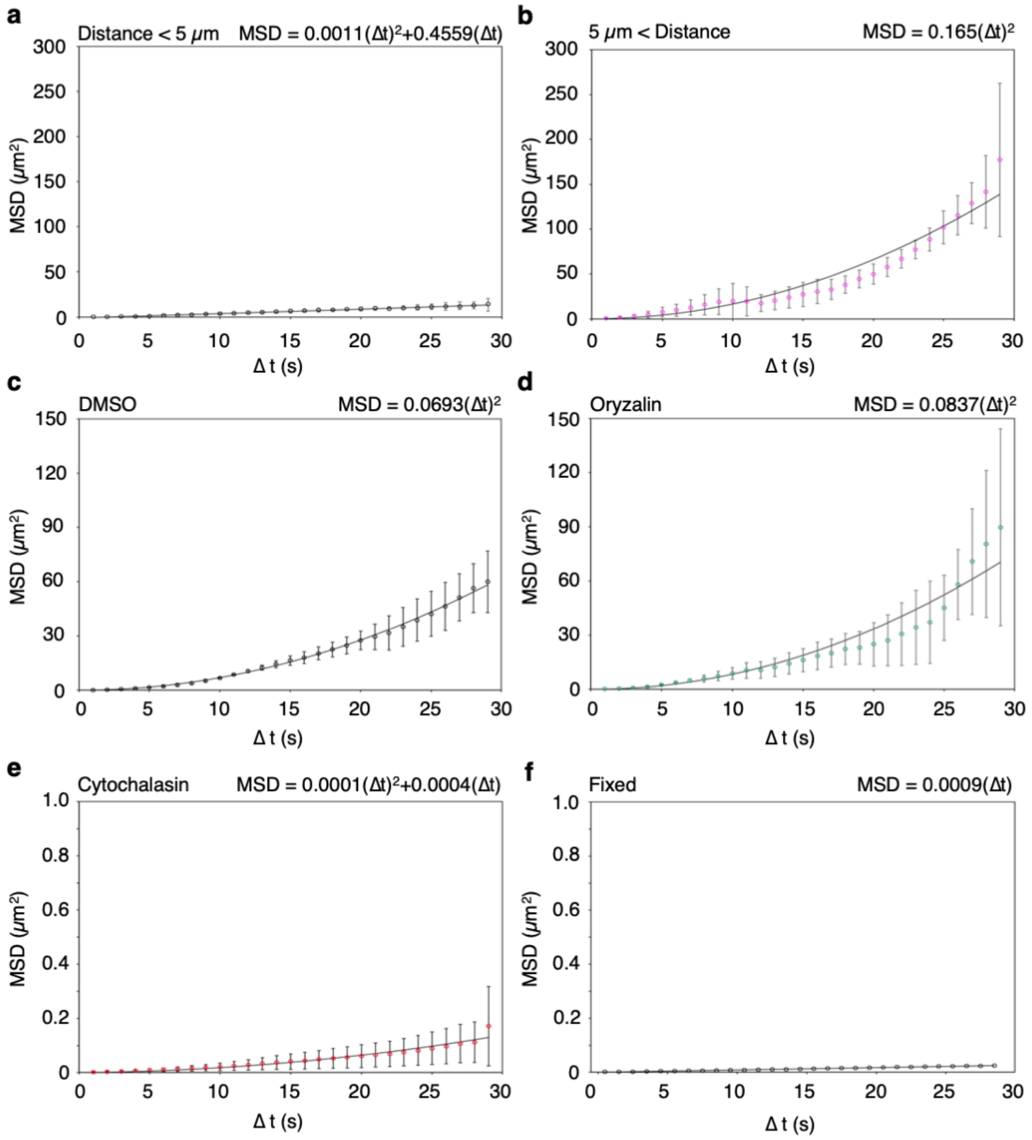

Supplementary Fig. 11 MSD analysis of mitochondrial movement. a-f Each plot are fitted to liner or curve models using the least-squares method with the Eq 2. **a, b** The mean squared displacement (MSD) of mitochondrial movements, which are separated to shorter (open circles) or longer (filled-magenta circles) than 5.0 μm distance. **c-e** MSD analysis for mitochondrial movement in DMSO-, oryzalin-, and cytochalasin-treated cells. **f** MSD analysis for mitochondria movement in fixed cell.

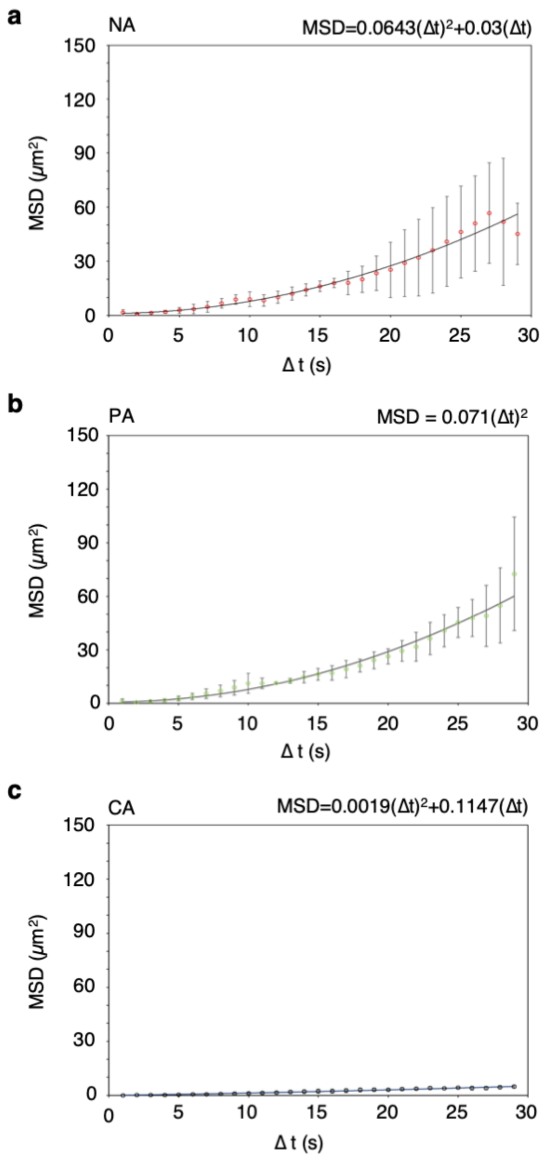

Supplementary Fig. 12 MSD analysis of mitochondrial movement associating with or without chloroplast. Each plot is fitted to curve models using the least-squares method with the Eq 2. **a-c** The mean squared displacement (MSD) analysis of three types of mitochondrial movements; no association (NA; **a**), partial association (PA; **b**), and continuous association (CA; **c**) with chloroplasts.

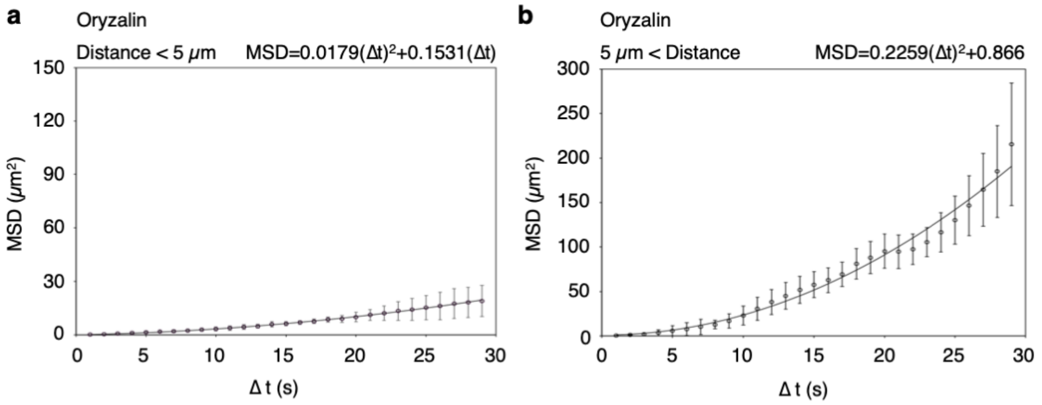

Supplementary Fig. 13 MSD analysis of mitochondrial movement in oryzalin-treated cell. Each plot is fitted to curve models using the least-squares method with the Eq 2. **a**, **b** The mean squared displacement (MSD) of mitochondrial movement in oryzalin-treated cells, which are separated to shorter (**a**) or longer (**b**) than 5.0 µm-migrate distance.

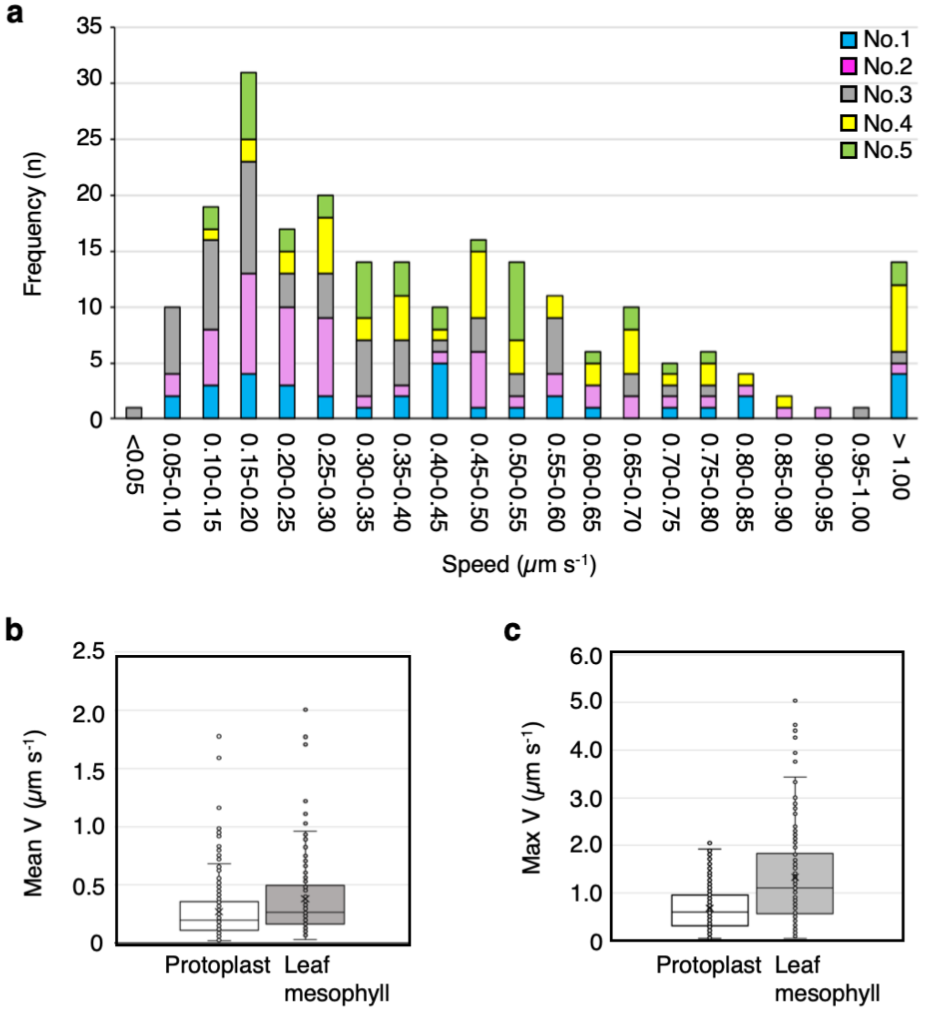

Supplementary Fig. 14 Comparison of speeds of mitochondrial movement between protoplast and leaf mesophyll cells. **a** Speed distribution of mitochondrial movement from five independent leaf mesophyll cells (No.1, blue, n=34; No. 2, magenta, n=45; No. 3, gray, n=57; No. 4, yellow, n=46; and No. 5, green, 64). **b, c** Mean (**b**) and maximum (Max) (**c**) velocity (V) of mitochondria in leaf mesophyll protoplasts and leaf mesophyll cells are shown as box plots.

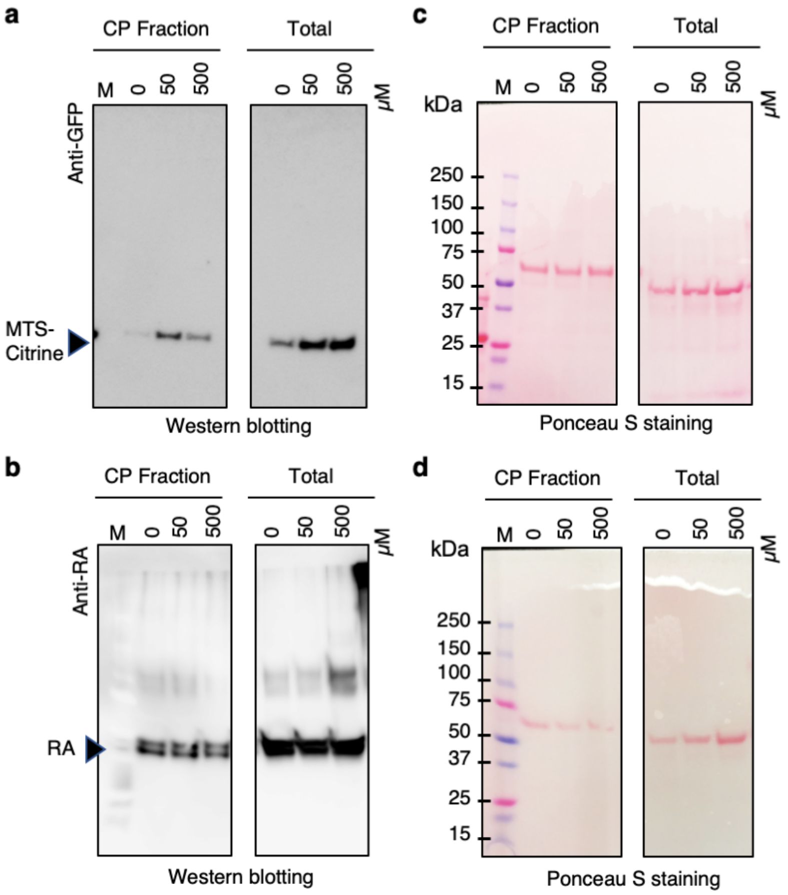

Supplementary Fig. 15 Co-isolation analysis of mitochondria and chloroplasts. a, **b** Western blot analysis of the isolated-chloroplast fraction (left) or total-leaf extract (right) using antibodies against GFP (**a**) or RuBisCO activase (RA) (**b**). MTS-Citrine at 28kD (**a**) and RA at 45kD (**b**) (arrow heads) are detected in both CP fraction and total leaf extraction from 0 µM, 50 µM and 500 µM cytochalasin-treated cells. **c**, **d** The images (**c**, **d**) show the equal loading of western blotting proteins (**a**, **b**) after transferred onto the PVDF membrane and stained with the Ponceau S solution.

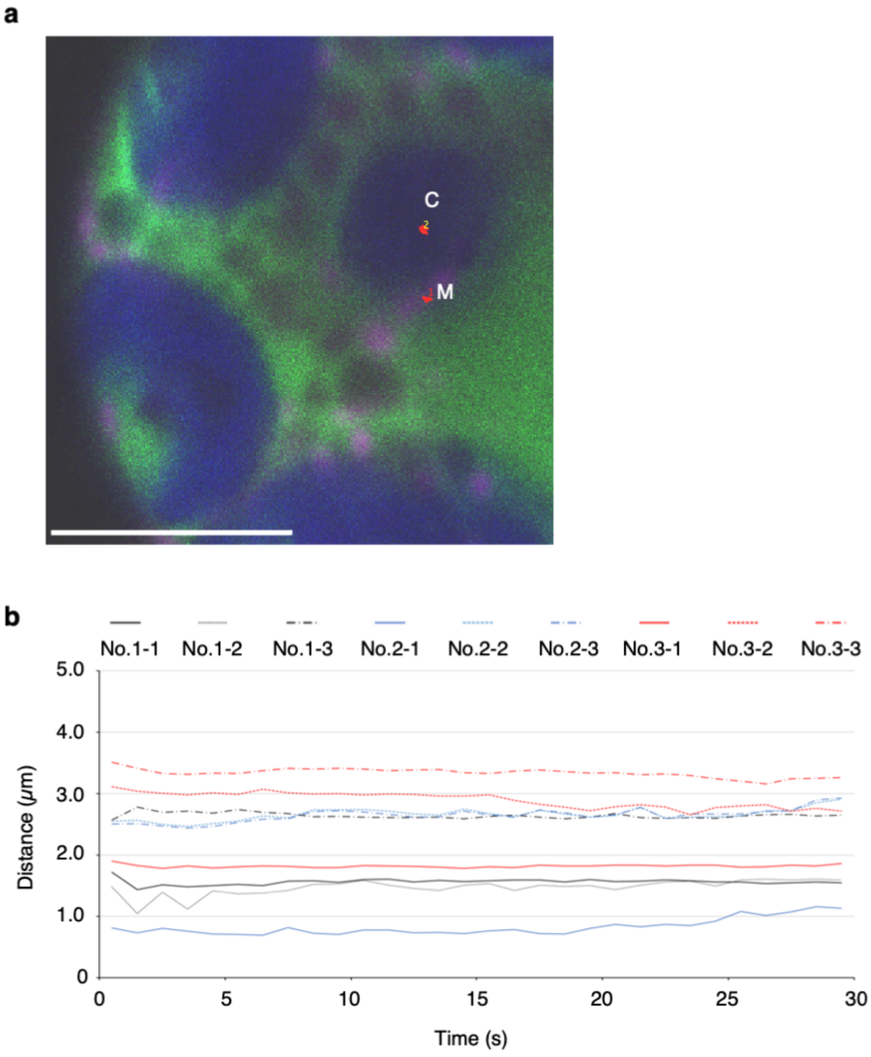

Supplementary Fig. 16 Interaction between mitochondrion and chloroplast in F-actin-disrupted protoplasts. a Representative image of trajectories of mitochondria (magenta) and chloroplast (blue) in a cytochalasin-treated protoplast, which expresses pGWT35S-Lifeact-Citrine gene (green). Time-lapse images were acquired for 30 s at 1-s intervals. Trajectory within 30 s of centroid of chloroplast (C) and mitochondrion (M) are shown as red color. Scale bar; 5 µm. **b** Distance between centroid of mitochondria and chloroplasts at each time points in 30 s are shown as lines. Each three set of chloroplast and mitochondrion (1-3) from three protoplasts (No. 1-3) is shown as different colors and lines (No. 1-1 to No. 3-3).

Supplementary Table 1. F-actin-independent association between mitochondria and chloroplasts. Number of mitochondria with or without actin filaments (FA) on chloroplasts in cytochalasin B-treated protoplasts.

	With FA	Without FA	Total
**DMSO**	146	122	268
**50 µM CB**	15	280	295
**500 µM CB**	15	206	221

Supplementary Table 2. Number of three types of mitochondrial movements associated with or without chloroplasts in three protoplasts. NA; no association, PA; partial association, and CA; continuous association.

Protoplast	NA	PA	CA	Total
**No.1**	24	57	37	118
**No.2**	25	46	64	135
**No.3**	9	33	38	80
**Sum**	58	136	139	333

Supplementary Table 3. Number of three types of mitochondrial movements associated with or without chloroplasts in leaf mesophyll cells. NA; no association, PA; partial association, and CA; continuous association.

Leaf cells	NA	PA	CA	Total
**No. 1**	3	13	20	36
**No. 2**	5	25	16	46
**No. 3**	2	36	28	66
**No. 4**	7	14	40	61
**No. 5**	3	10	30	43
**Sum**	20	98	134	252

Supplementary Table 4. Parameter of MSD analysis and character of mitochondrial movements. *D*: Diffusion coefficient, *v*: mean velocity, MD: migrate distance, NA: No association with chloroplast, PA: partial association with chloroplast, CA: continuous association with chloroplast, Dire: Direct, and Diff: Diffusion. The chi-squared value, χ^2 and p-value to test the goodness of fit about the MSD analysis fits are shown.

	D (µm^2 s^{-1})	v (µm/s)	Pattern	$\chi 2$	P − value
**MD < 5 µm**	0.11	0.0332	Dire + Diff	1.00	0.317
**5 µm < MD**	-	0.406	Dire	6.69E-05	0.993
**DMSO**	-	0.263	Dire	1.00	0.317
**Oryzalin**	-	0.289	Dire	0.419	0.517
**MD < 5 µm (Oryzalin)**	0.038	0.134	Dire + Diff	1.00	0.317
**5 µm < MD (Oryzalin)**	-	0.475	Dire	0.931	0.334
**Cytochalasin**	0.00010	0.0100	Dire + Diff	1.00	0.317
**NA**	0.00075	0.254	Dire + Diff	1.00	0.317
**PA**	-	0.267	Dire	1.00	0.317
**CA**	0.029	0.0436	Dire + Diff	0.587	0.444
**Fixed**	0.00023	-	Brownian	1.00	0.317

Supplementary Table 5. Number of mitochondria associated with or without chloroplast in 500 μM CB-treated protoplasts. SD: standard deviation. Student's t-test (With chloroplast vs Without chloroplast): $1.341E^{-19}$

No. Protoplast (n)	With chloroplast (%) (SD)	Without chloroplast (%) (SD)	Total No. Mitochondrion (n)
**10**	92.8 (5.1)	8.1 (3.5)	726
