# Supplementary material for: Mitochondrial movement during its association with chloroplasts in Arabidopsis thaliana
Source: Commun Biol. 2021 Mar 5;4:292. doi: 10.1038/s42003-021-01833-8 (PMC7935954; doi:10.1038/s42003-021-01833-8)
Supplement: Supplementary file 3 — Description of Supplementary Files [file 42003_2021_1833_MOESM3_ESM.pdf]

Description of Additional Supplementary Files

File name: Supplementary Movie 1

Description: Mitochondrial movement in a protoplast. Time-lapse images of mitochondria (green) and chloroplasts (magenta) observed by confocal laser-scanning microscopy. The number at the top right indicates the time in seconds.

File name: Supplementary Movie 2

Description: Mitochondrial movement in a protoplast. Time-lapse images of mitochondria (green) and chloroplasts (magenta) observed by confocal laser-scanning microscopy with trajectories of mitochondrial movements. The number at the top right indicates the time in seconds.

File name: Supplementary Movie 3

Description: Mitochondrial movement in an oryzalin-treated protoplast. Time-lapse images of mitochondria (green) and chloroplasts (magenta) observed by confocal laser-scanning microscopy. The number at the top right indicates the time in seconds.

File name: Supplementary Movie 4

Description: Mitochondrial movement in an oryzalin-treated protoplast. Time-lapse images of mitochondria (green) and chloroplasts (magenta) observed by confocal laser-scanning microscopy with trajectories of mitochondrial movements. The number at the top right indicates the time in seconds.

File name: Supplementary Movie 5

Description: Mitochondrial movement in a cytochalasin-treated protoplast. Time-lapse images of mitochondria (green) and chloroplasts (magenta) observed by confocal laser-scanning microscopy. The number at the top right indicates the time in seconds.

File name: Supplementary Movie 6

Description: Mitochondrial movement in a cytochalasin-treated protoplast. Time-lapse images of mitochondria (green) and chloroplasts (magenta) observed by confocal laser-scanning microscopy with trajectories of mitochondrial movements. The number at the top right indicates the time in seconds.

File name: Supplementary Movie 7

Description: Wiggling of mitochondria associated with a chloroplast in a cell treated with 50 μM cytochalasin B. Wiggling of mitochondria (arrow) associated with a chloroplast. The number at the top right indicates the time in seconds.

File name: Supplementary Movie 8

Description: Wiggling of mitochondria associated with a chloroplast in a cell treated with 500 μM cytochalasin B from MTS-Citrine transgenic A. thaliana. Wiggling of mitochondria (arrow) associated with a chloroplast. The number at the top right indicates the time in seconds.

File name: Supplementary Movie 9

Description: False mitochondrial movement (fluorescence fluctuation) in a fixed cell. Time-lapse images of fixed mitochondria (green) observed by confocal laser-scanning microscopy. Only false movement due to fluorescence fluctuation could be observed. The number at the top right indicates the time in seconds.

File name: Supplementary Movie 10

Description: Wiggling of mitochondria associated with a chloroplast. Cropped video from Supplemental Movie 1 showing wiggling of mitochondria (arrow) associated with a chloroplast. The number at the top right indicates the time in seconds.

File name: Supplementary Movie 11

Description: Mitochondrial movement in a leaf palisade mesophyll cell. Time-lapse images of mitochondria (green) and chloroplasts (magenta) observed by confocal laser-scanning microscopy. The number at the top right indicates the time in seconds.

File name: Supplementary Movie 12

Description: Interaction between mitochondrion and chloroplast in F-actin-disrupted protoplast. Time-lapse images of a leaf protoplast expressing pGWT35S-Lifeact-Citrine gene (green) were acquired for 30 s at 250-ms intervals by confocal laser-scanning microscopy. The centroid of mitochondria (magenta) and chloroplast (blue) were shown as red color.

File name: Supplementary Data 1

Description: Source data underlying plots shown in figures.
